# Isogyres – Manifestation of Spin-orbit interaction in uniaxial crystal: A closed-fringe Fourier analysis of conoscopic interference

**DOI:** 10.1038/srep33141

**Published:** 2016-09-14

**Authors:** C. T. Samlan, Dinesh N. Naik, Nirmal K. Viswanathan

**Affiliations:** 1 School of Physics, University of Hyderabad, Hyderabad 500046, India

## Abstract

Discovered in 1813, the conoscopic interference pattern observed due to light propagating through a crystal, kept between crossed polarizers, shows isochromates and isogyres, respectively containing information about the dynamic and geometric phase acquired by the beam. We propose and demonstrate a closed-fringe Fourier analysis method to disentangle the isogyres from the isochromates, leading us to the azimuthally varying geometric phase and its manifestation as isogyres. This azimuthally varying geometric phase is shown to be the underlying mechanism for the spin-to-orbital angular momentum conversion observed in a diverging optical field propagating through a z-cut uniaxial crystal. We extend the formalism to study the optical activity mediated uniaxial-to-biaxial transformation due to a weak transverse electric field applied across the crystal. Closely associated with the phase and polarization singularities of the optical field, the formalism enables us to understand crystal optics in a new way, paving the way to anticipate several emerging phenomena.

The discovery in 1813 by David Brewster that uniaxial crystals, when illuminated with white light and observed between crossed polarizers shows a system of colored rings intersected by a rectangular black cross^1^ still continues to challenge our understanding of this more than two centuries old optical phenomenon. A large number of classic and modern books and review articles are written, dedicated to homogeneous non-magnetic optical crystals and the interaction of light with it^2^,^3^,^4^,^5^,^6^,^7^,^8^,^9^,^10^,^11^,^12^. Notwithstanding, new phenomena, broadly classified under spin-orbit interactions (SOI) of light^13^ in anisotropic optical media continues to make their way into as one of the actively researched areas. This includes passive and active methods of generation of scalar and vector singular beams using uniaxial crystals via spin-to-vortex conversion^14^,^15^,^16^,^17^,^18^,^19^ and its dynamics^20^,^21^, unfolding of a uniformly polarized optical vortex beam propagating through a birefringent crystal^22^ and more recently theoretical study to understand orbital angular momentum (OAM) sidebands by breaking the rotational symmetry of a uniaxial crystal via Pockels effect^23^.

The images observed by Brewster, known as a conoscopic interference pattern, are formed when an anisotropic crystal is viewed under convergent/divergent cone of light and is due to the refractive index variation with the direction of incident light, represented by an ellipsoid called indicatrix^24^. Observing the uniaxial crystal cut perpendicular to the optic-axis (OA) the interference pattern shows circular symmetry centred around the OA. The ordinary refractive index *n*_*o*_ tangent to the concentric circles and the extraordinary index *n*_*e*_ perpendicular to it leads to two *k*-surfaces respectively with azimuthal and radial eigen polarization^12^. When the fast and slow optical waves, due to *n*_*o*_ and *n*_*e*_ of the crystal, are resolved into components in the same direction by the analyzer, they interfere with each other causing colored interference fringes when illuminated with white light or a set of monochrome rings when a laser source is used. This pattern is known as isochromates for obvious reasons. In addition, we also observe a dark cross known as ‘Maltese cross’ corresponding to the direction of the polarizer and the analyzer, along which the state of polarization (SoP) of the beam passing through the crystal does not change. Known as isogyre, the two isogyres, perpendicular to each other, intersect at the center of the visual field, coinciding with the OA of the uniaxial crystal. Brought to light by Berry *et al*., the black ‘*fermion brush*’ (isogyres) crossing the ‘bullseye’ appearing in the conoscopic interference pattern of a biaxial material has its origin in the geometric phase (GP) of each polarization accumulated in a circuit around the OA^25^. For a uniaxial crystal, the brush condition is satisfied four times in a circuit around the OA resulting in the black cross pattern.

These characteristic features of the conoscopic interference pattern have been used extensively to study the anisotropic properties such as linear/circular birefringence and dichroism, optical activity and optical inhomogeneity of materials such as crystals, organic tissues, polymers and strained glasses^26^,^27^,^28^ and is also a critical tool in identifying minerals^29^. In most of these applications, the isochromates are commonly used to measure the optical properties of materials and their changes. The isogyres, with fairly complicated structure^30^,^31^, on the other hand are seen to weaken the interference pattern, thought to be a nuisance and as they are entangled with the isochromates are found to hinder the measurement accuracies. It was widely believed that the isochromates possess more physical features than the isogyres and so the need to eliminate the isogyres from the conoscopic pattern lead to the development of a number of experimental techniques to study the crystal properties^32^,^33^,^34^,^35^,^36^.

It is known that a cyclic transformation of the SoP using stacked birefringent wave plates leads to the accumulation of GP^37^. Our recent experimental investigation revealed a singular behavior in the topology of GP acquired by the final state of the optical field in response to the rotation of wave plates introduced between crossed polarizers^38^. From the insight gained, an investigation of the conoscopic pattern is carried out to understand the origin, manifestation and manipulation of geometric phase in a z-cut birefringent crystal. In this sense, the core theme of the present research is to explore the origin of isogyres which was given less importance compared to isochromates in many studies on optical crystals.

To connect the information contained in the conoscopic interference pattern with the new approach involving the GP and topological singularities^39^, we propose and apply closed-fringe Fourier analysis (CFFA) method^40^,^41^. Using this technique the isochromates and isogyres are first separated without loosing any of the intricate features involving the dynamic and geometric phase acquired by a focused Gaussian beam propagating along the OA of a uniaxial crystal. Application of weak transverse electric field across the crystal breaks its symmetry, resulting in a uniaxial-to-biaxial transformation, initiated by weak optical activity in the crystal. All these features are captured by the CFFA technique and will be useful to explain the phase and polarization singularities in the beam and the formation of Airy spiral apart from gaining a deeper understanding of the SOI in optical crystals, connecting them to conical intersections^42^.

The schematic diagram of our experimental setup, designed for obtaining the conoscopic interference pattern from a uniaxial crystal, is shown in Fig. 1. It comprises two Glan-Thompson polarizers P_1_ and P_2_ for orthogonal projection of the polarization state, a telescopic lens system with lenses L_1_ and L_2_ for focusing the beam on the crystal and to collimate it back followed by a CCD camera to record the conoscopic interference pattern. The polarizer (P_1_) prepares horizontally polarized light beam from the laser and the polarizer (P_2_) projects the beam to its orthogonal state. Potassium dihydrogen phosphate (KDP) crystal, a negative uniaxial and electro-optic material belonging to 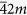 class tetragonal crystal is introduced between the two polarizers. The KDP crystal is 5 cm long, 1 cm wide and is *z*-cut. The principal refractive indices are *n*_0_ = 1.5079 and *n*_*e*_ = 1.4673 at the laser wavelength of 632.8 *nm*. The lens L_1_ (*f* = 1.2 cm) focuses the incoming beam and the lens L_2_ (*f* = 3 cm) collimates it back. We insert the KDP crystal at the focal plane of L_1_ and align it such that the OA and the beam axis coincide. We record the interference pattern arising due to the orthogonal projection using the CCD camera without applying any voltage across the crystal (Fig. 2a). The recording is repeated subsequently by applying a voltage of 10^2^ V across the crystal, in the *x*-direction generating an electric field of 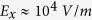 inside the crystal. The non-vanishing electrooptic coefficients 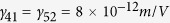 change the principal axis direction as well as the principal refractive indices due to Pockels effect, a manifestation of first-order nonlinearity in the polarization of the crystal. As a result, the index ellipsoid of the crystal transforms from uniaxial 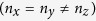 to biaxial 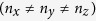 as given below,


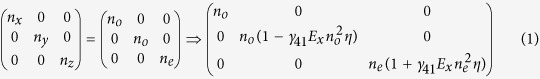


where, 
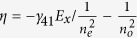
.

The conoscopic interference pattern obtained using the experimental setup shown in Fig. 1 comprises of isochromates, the concentric bright and dark circular rings and isogyres, the dark cross as shown in Fig. 2(a). The circularly symmetric conoscopic pattern can thus be considered as a product of two interference patterns, respectively the radially invariant isogyres and azimuthally invariant isochromates. The symmetric pattern gets distorted upon application of transverse electric field resulting in non-zero intensity along the OA, and the cross pattern becomes hyperbolic with the circular rings becoming slightly elliptical, as can be seen from Fig. 2(b).

## Theoretical formalism

Consider a uniaxial crystal of length *d* with its OA parallel to the z-axis of laboratory frame with 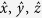 as the Cartesian coordinates. Let us theoretically formulate the effect of birefringence experienced by a plane-wave 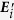 during its propagation through the crystal. The propagation vector *k* of 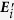 is assumed to be oriented along 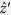, the z-axis of the plane-wave frame with 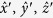 as its coordinates. With 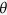 as the angle between 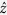 and 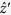, this leads to the projection of OA in the 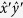 plane. Due to the ordinary (*n*_0_) and the extraordinary 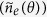 refractive index ellipsoid of the crystal as represented in Fig. 3(a), the field components that are parallel and orthogonal to the projected OA acquire a phase difference 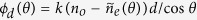 between them. Additionally, it can also be shown that the orientation of the projected OA in the 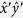 plane makes an angle 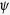 with 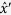 axis.

To obtain the transformation of the optical field of the plane wave due to its propagation through the crystal, we project the 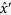 and 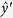 components of the initial field 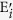 onto the directions parallel and orthogonal to the projected OA using a rotation matrix 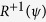. We introduce the phase difference 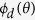 through unitary matrix 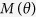 and finally project the field components back to 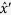 and 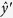 directions using an inverse rotation matrix 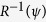 to obtain the final field 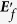. The transformation equation can be written as,


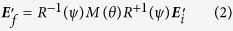


where, 
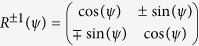
 and 
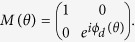


To investigate the spin-to-orbit angular momentum conversion resulting from the phase modulation acquired by an optical beam after passing through the crystal, we probe the transformation using the optical field in σ^+^ (right) and σ^−^ (left) circular polarization basis. Following a straight forward matrix algebra, the optical field at the output of the crystal, corresponding to the initial field 
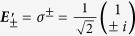
 is written as,


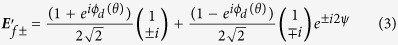


It should be noted that the SoP in the first-term of the equation is the same as that of the initial field with the acquired phase difference accounted as the dynamic phase 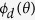, a consequence of the crystal anisotropy. The second-term in the equation represents the SoP orthogonal to the initial field. With the flipping of polarization handedness, the acquired geometric phase 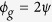 is observed apart from the accumulation of the dynamic phase. Considering a variable retarder, the fraction of spin-flip depends on 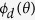 with 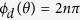 yielding zero and 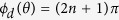 yielding 1 where *n* is an integer. A diverging beam can be considered as a collection of plane-waves with a range of *θ*. The central plane wave in the beam, corresponding to *θ* = 0, lies along the OA of the crystal (*z*-axis). As shown in Fig. 3(b), the rest of the plane-waves in the beam propagate through the crystal in slightly different directions, *i.e.*, with *θ* ≠ 0 and acquire the dynamic and geometric phases as described by Equation (3). The rotational invariance of the inclination angle *θ* around the optic-axis results in azimuthal variation in the projection angle 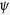. In other words, as shown in Fig. 3(c), as the inclination angle *θ* becomes a function of the radial coordinate 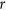 with *r* ∝ tan(*θ*), the projection angle 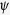 becomes equal to the azimuthal coordinate *φ*^43^,^44^.

With this insight, we write Equation (4) that describes the spatially varying final field for circularly polarized diverging beam:


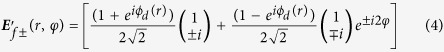


The equation reveals that the dynamic phase is a function of *r*, the radial coordinate, whereas the geometric phase is a function of *φ*, the azimuthal coordinate. Since 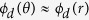 this results in spatial modulation as 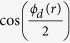 and 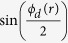, the amplitudes of the two circular polarization basis in Equation (4) and the fraction of spin-flip in this case is limited to a maximum value of 0.5.

The phase factor 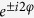 in the polarization orthogonal to the initial field describes a helical wavefront with charge *l* = ±2, indicating the presence of orbital angular momentum. The total angular momentum of the optical beam is the sum of spin (*σ*) and orbital angular momentum (*l*). For 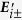 depending on the choice of circular basis, *σ* = ±1, the total angular momentum (*j* = σ + *l*) can be ±1. Considering the situation of 50% spin-flip, we can write the total angular momentum for 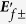 as 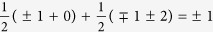 where the numbers in the brackets represent *σ* and *l* values of the two terms in Equation (4). The conservation of the total angular momentum for the beam can be attributed to the rotational symmetry exhibited by the inclination angle *θ* around the OA of the crystal. Now the application of an external electric field across the crystal can break the symmetry through uniaxial-to-biaxial transformation resulting in non-conservation of total angular momentum.

**Figure 1:**
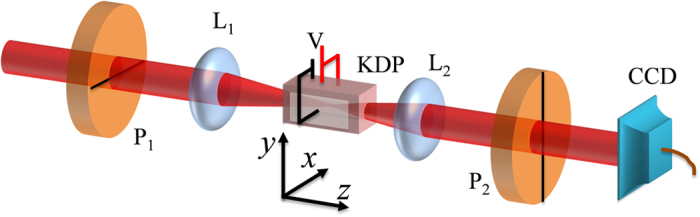
Experimental setup. (**a**) Polarizers (P_1_ and P_2_) are oriented in horizontal (parallel to x-axis) and vertical (parallel to y-axis) directions; lens, L_1_ is used to converge the beam and lens L_2_ is used to collimate it back.

Considering Gaussian envelope for the diverging optical field, the first and second terms in Equation (4) represent a Gaussian and Laguerre-Gaussian (LG) (with *l* = ±2) modes, respectively. To model the experimental setup shown in Fig. 1, where a horizontally polarized diverging Gaussian beam propagated through the crystal is projected to its orthogonal (vertical) polarization resulting in the formation of isogyre, we simplify the transformation equation (Equation 4) in the circular basis by exciting both circular polarization states simultaneously. The initial state given by


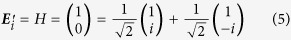


then transforms to


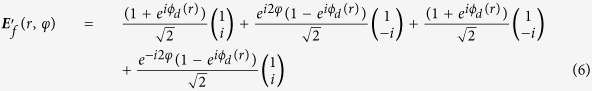


The output beam intensity after projecting on to vertical polarization state resembles an interference equation given by


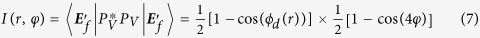


where 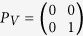 is the projection operator for vertical polarization state. Equation (7) represents the recorded interference pattern, which is a product of two terms which are functions of *r* and 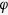 respectively representing *isochromates*, 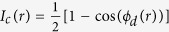 having contributions from dynamic phase and *isogyres*, 
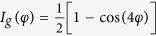
 having contributions from geometric phase acquired by the beam field propagating through the crystal. It is important to note here that Equation (7) is the same as that derived by Berry *et al*., for a more general case [Equation (31) in ref. 25].

The multiplication of the terms 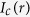 and 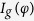 in the interferogram enables us to write the Fourier transform of the interference pattern as a convolution between the individual Fourier spectra of 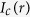 and 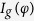 as follows,


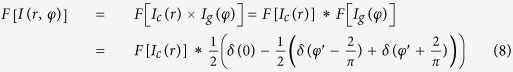


where, 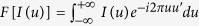 is the Fourier transform of a function 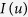 with *u* as the coordinate. Here *u*′ is the coordinate of the Fourier spectrum, *δ* is the well-known Dirac delta function and the symbol ‘*’ stands for the convolution operator. The spectrum corresponding to isochromates 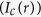 can be assumed to be up-chirped due to decreasing spacing between the concentric rings of isochromates. On the other hand, the spectrum corresponding to isogyres can be assumed to be a delta function as the isogyres bands are periodic^37^.

## Results

**Figure 2:**
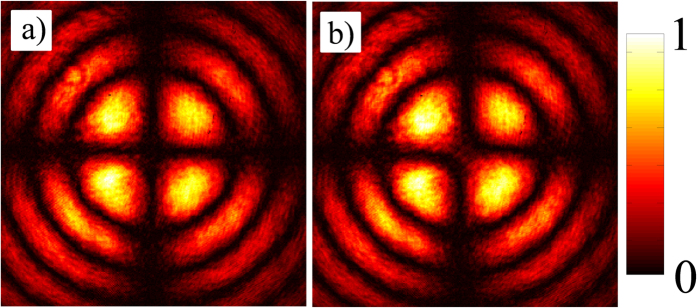
Conoscopic interference fringes of the KDP crystal without (**a**) and with (**b**) applied electric field.

**Figure 3:**
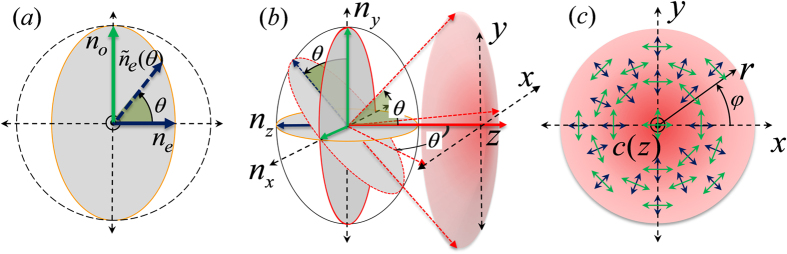
Refractive index ellipse of a uniaxial crystal and its *θ* dependent birefringence is shown (**a**). Associated index ellipsoid (**b**) with a different plane of propagation specified by *θ* and the rotation of ordinary (green line) and extra-ordinary (violet line) polarization modes around the optic-axis are illustrated in (**c**).

We attempt to address the origin of the conoscopic interference pattern from the viewpoint of its association with the dynamic and geometric phases acquired by the optical field while passing through the uniaxial crystal and connecting them to optical singularities. The separation of the isochromates and the isogyres to extract their phase and intensity distribution is carried out using the closed-fringe Fourier analysis (CFFA) described in method section. It should be noted that the CFFA provides information regarding the phase difference between the interfering beams leading to the characteristic interference fringe pattern; and not the phase map of the optical field whose intensity is represented in the recorded images. In this sense, the phase extracted by our method provides access to the phase difference between the two orthogonal components of the optical field that was made to interfere though the projection operation by the polarizer P_2_. This allows us direct access to the optical information and processes unfolding in the crystal in response to a physical process. To realize this, we first process the conoscopic interference fringes shown in Fig. 2(a). Subsequently extend the method to study the influence of the weak transverse electric field, 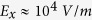 applied across the crystal (Pockels effect), resulting in Fig. 2(b), to understand its effect on the optical phases. It is important to point out here that the change in induced birefringence due to transverse electrooptic effect in KDP crystal is orders of magnitude smaller than its longitudinal counterpart^45^. Nevertheless, our CFFA technique is sufficiently sensitive to map the crystal’s transition from uniaxial-to-biaxial and rotation of the singularity axes plane by 45° due to associated optical activity^45^,^46^. The crystal is uniaxial, with zero retardation, only for light traveling along the OA and when the applied voltage is zero. However, the linearly polarized light, even traveling along the OA, becomes weakly elliptical and pass through the polarizer P_2_, as can be seen from Fig. 2(b), when a voltage is applied. As the coupling between the Pockels effect and optical activity is very small for applied electric field of *10*^*4*^ *V/m*^47^, the CFFA based method of disentangling the isochromates and isogyres is valid.

**Figure 4:**
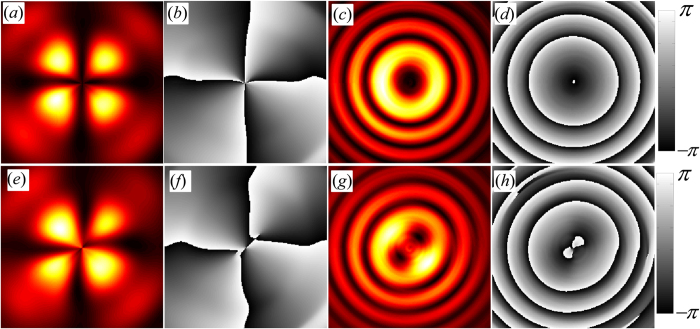
Intensity pattern of the isogyres and isochromates (**a**,**c**) and the corresponding wrapped phase maps (**b**,**d**) separated from the consoscopic pattern shown in Fig. 2(a), without applied electric field. The corresponding intensity and phase maps in the presence of transverse electric field are shown in (**e**–**h**).

The intensity and phase information of the isogyres and isochromates separated using the CFFA, following the procedure outlined in Methods section, are shown in Fig. 4. The intensity plots shown in Fig. 4(a, c, e and g) are normalized whereas the wrapped phase maps (*b*, *d*, *f* and *h*) shown in gray-scale have the range of −*π* to *π*. The images shown in the top row of Fig. 4 correspond to the information extracted for the case when no electric field is applied across the crystal. The intensity image shown in Fig. 4 (a) corresponds to the isogyres of Fig. 2(a) with the corresponding phase map in Fig. 4(b) which shows a singularity at the center. As mentioned earlier, this is a conical singularity of the two refractive index sheets, with *n*_*+*_ = *n*_*−*_ and the contours around them form closed loops^37^.

Around the singularity, the phase map shows an azimuthal variation of 8*π* representing an associated charge of *l* = 4. As mentioned earlier, the phase map represents the phase difference between the two orthogonal components of optical field emerging from the crystal superposed through the projection by P_2_. The 8*π* azimuthal variation of the phase indicates that the isogyres are formed due to the superposition of two doubly but oppositely charged Laguerre-Gaussian modes. On other hand, the phase map of isochromates (Fig. 4(d)) does not exhibit any singular behavior and shows a quadratic phase variation, corresponding to the phase difference that the superposed orthogonal components acquire during the conical propagation through the crystal due to *n*_*o*_ and *n*_*e*_.

**Figure 5:**
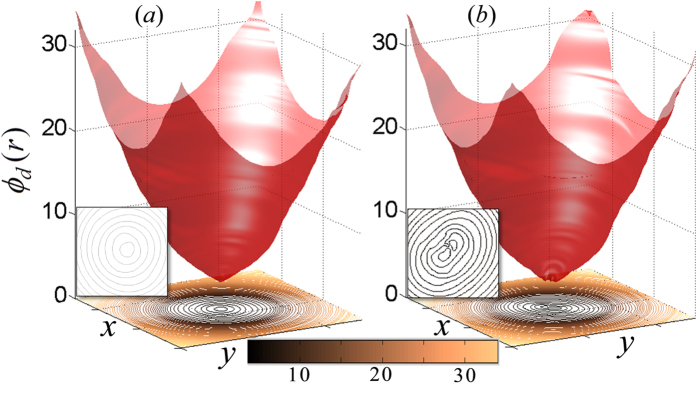
Unwrapped phase map (in radians) of isochromates without (**a**) and with (**b**) applied electric field. The corresponding contour lines are shown on the floor of the image, and the region of interest is highlighted in the inset.

The intensity image shown in Fig. 4(e) represents the isogyres separated from the conoscopic pattern shown in Fig. 2(b) when a weak electric field is applied across the crystal. The corresponding phase map is shown in Fig. 4(f) from which we can clearly see that the isogyres are split and drift away from the singular point when the Pockels effect is turned on. The presence of Pockels effect perturbes the OA and its surrounding by splitting the singularity corresponding to *l* = 4 into two *l* = 2; a manifestation of the transformation of the crystal from uniaxial to biaxial. The corresponding changes in the isochromates are also clearly visible in the Fig. 4(g,h) where the circular symmetry is broken, and two spots corresponding to the biaxial crystal axes are seen. Unwrapping of the phase maps of the isochromates shown in Fig. 4(d,h) reveals a paraboloidal structure as shown in Fig. 5(a,b) respectively without and with applied electric field. The paraboloidal structure represents the stereographic projection of the refractive index ellipsoid and reveals the birefringence of the crystal^25^. The single minimum of the paraboloid transforms to double minima due to Pockels effect upon the application of electric field. The contour plots highlighting this transformation are given in inset of Fig. 5, which shows the 45° orientation of the elliptical contours (Fig. 5(b)) due to the field induced optical activity in the KDP crystal.

## Discussion

The orthogonal projection of a state after its interaction with a system provides critical topological information about the system^48^. This powerful technique based on the orthogonal projection of a state is utilized to investigate the crystal properties through its interaction with the optical field by designing the conoscopic experiment. Our investigations reveal that the isochromates results from the accumulation of the dynamic phase and the isogyres are due to geometric phase accumulation through the interaction with the crystal. In this sense, our results describe a fundamental aspect of the evolution of optical field in a crystal.

For a circularly polarized diverging optical field propagating through a z-cut crystal, flipping of spin is observed for a part of the total field. This component is shown to acquire a geometric phase that is a function of the azimuthal coordinate in the observation plane, due to the conical (converging/diverging) nature of the optical beam inside the crystal. The accumulation of azimuthally varying phase leads to a redistribution of energy of that field component similar to a Laguerre-Gaussian (LG) beam.

The metasurfaces with location-dependent OA oriented in an azimuthal fashion working on the Pancharatnam-Berry phase principle are known to convert a circularly polarized Gaussian beam to a Laguerre-Gaussian (LG) beam^49^ or an elliptically polarized light beam into a vector beam with any desired polarization distribution^50^,^51^. In the case of Q-plate as well, with 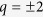, it is the azimuthally varying orientation of OA that introduces the geometric phase required for spin-to-orbital angular momentum conversion^52^. However, since both the metasurface and Q-plate have an azimuthally varying orientation of the OA, it requires only a plane-wave with an inclination angle 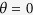 to acquire the azimuthally varying geometric phase. In the process, the beam acquires only a constant value, 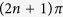, as the dynamic phase. This condition ensures a complete spin-flip with a transfer of change in spin angular momentum 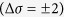 to the orbital part. In the case of the uniaxial crystal, the optic-axis is location independent and oriented along z- direction. The azimuthal variation of orientation of OA projections is achieved through radially varying propagation vectors in the diverging optical beam. The resultant dynamic phase acquired by the optical beam being a radial function, the maximum achievable spin-flip and conversion to orbital angular momentum is limited to 50%.

Though Q-plates and metasurfaces offer flexibility in the design of angular momentum convertors in terms of charge values *l*, the devices are wavelength-specific as the value of 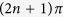 for the dynamic phase to achieve the total angular momentum conversion is satisfied only for a particular wavelength. The angular momentum conversion in the uniaxial crystal though is specific to charge value of 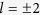 the modulation in the optical field due to the radially varying dynamic phase 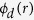 ensures that the conversion efficiency of 50% is satisfied for all the wavelengths. As a consequence, the uniaxial crystal has the potential for applications as achromatic orbital angular momentum generator.

By treating a linearly polarized Gaussian beam as a superposition of right and left circular polarization modes with zero initial phase difference propagating through the crystal with same divergence, each mode induces a counter-rotating geometric phase on the left and right circular polarization with charges 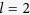 and 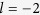, respectively. The azimuthal conoscopic pattern, the isogyres, is observed due to the interference between these two modes by projecting to a linear state, orthogonal to the input polarization state. Hence, the isogyres pattern can be considered as the manifestation of the geometric phase induced in the azimuthal direction and the resulting spin-to-orbit angular momentum conversion in the uniaxial crystal. This mechanism is also responsible for understanding phenomena such as polarization singularities of optical fields^24^, optical vortex generation^15^,^16^,^17^,^18^, Airy’s spiral^53^, etc. Important to point out that the analysis of field and polarization state topologies acquired due to propagation through a crystal from the viewpoint of optical vortices and polarization singularities was brought to the fore recently as due to the interplay between spin and orbital degrees of freedom^54^,^55^. In this context, our polarization-blind approach, accessing purely the conoscopic interference intensity pattern, though starts with less information for analysis but grants a unique access to the phase difference between the interfering field components that generates the conoscopic and polarization singularity patterns. In this sense, our approach with its distinct ability to capture the phase accumulation that unfolds prior to interference, allows us to go a step deeper into understanding the process by disentangling the phase contributions having geometric and dynamic origins.

## Method

**Figure 6:**
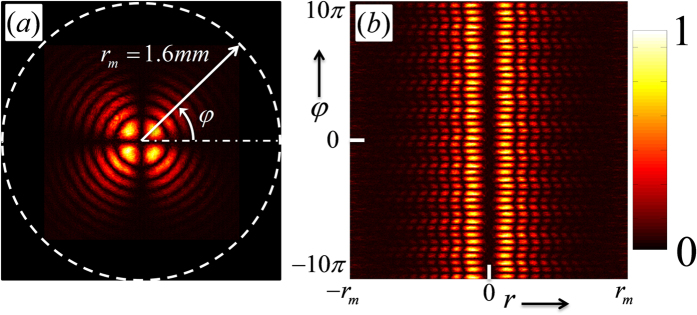
Conoscopic interference pattern (**a**) and its transformation to ‘extended’ cylindrical coordinate system (**b**).

Figure 6(a) shows the conoscopic interference pattern arising from the propagation of coherent, diverging Gaussian beam through the KDP crystal that is kept between two crossed polarizers. The interference pattern, recorded in *x*-*y* plane of the lab coordinate, comprises a set of concentric bright and dark rings that are termed as the isochromates and a dark cross band known as the isogyres. The dynamic and geometric phases are entangled in all real cases. The conoscopic interference pattern of crystals and QH polarization gadgets for geometric phase introduction are few examples in this category. However, the variable-separable form of the terms in Equation (7) hints that they can be separated in polar coordinates. We thus transform the image from the Cartesian lab coordinate system to ‘extended’ cylindrical coordinate system such that *r* varies from −*r*_*m*_ to *r*_*m*_ with *r*_*m*_ = 1.66 *mm* and 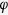 varies from 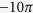 to 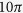. Unlike the conventional transformation from 0 to *r*_*m*_, the proposed transformation from −*r*_*m*_ to *r*_*m*_ allows extraction of critical information around *r* = 0 without discontinuity (hard-aperture) induced errors. Additionally, the variation of 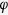 from 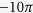 to 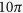 instead of conventional 0 to 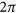, allows the separation of the spectral part corresponding to the isogyres from the central peak corresponding to DC content in the interference pattern. The resultant image is shown in Fig. 6(b). By doing this transformation, it can be seen that the fringes due to isochromates and isogyres are transformed into a mesh-like structure. There are no more closed loops or circular fringes after the transformation. This allows us to use the Fourier transform method of fringe analysis^40^ and attempt the extraction of the information from isochromates and isogyres separately. The spectral terms corresponding to 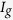 in Equation (8) comprises of three delta functions, one located at the origin and the other two are shifted by an amount 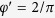 in positive and negative direction of the azimuthal axis.

**Figure 7:**
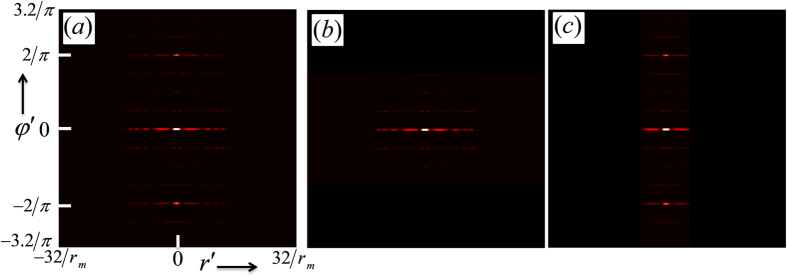
Absolute value of Fourier transform spectrum (**a**); part of spectrum corresponding to isochromates (**b**), part of spectrum corresponding to isogyres (**c**).

**Figure 8:**
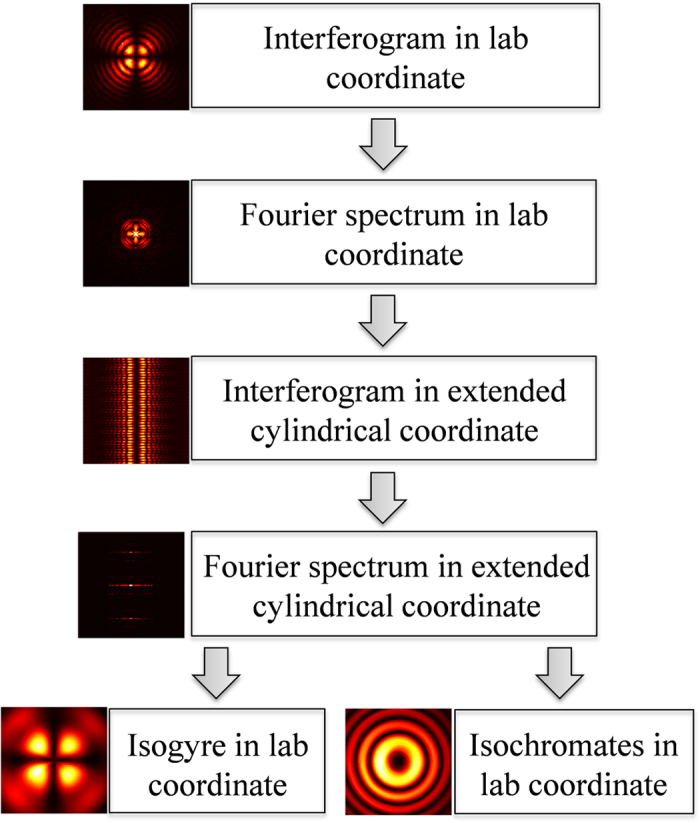
Scheme of the procedure. Images shown are corresponding outcomes of the process.

The absolute value of the two-dimensional Fourier transform of the intensity image shown in Fig. 2a is shown in Fig. 7a where, the coordinates 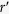 ranges from −32/*r*_*m*_ to +32/*r*_*m*_ and 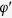 ranges from 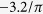 to 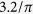. The spectral points arranged in the vertical 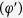 and horizontal 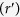 directions correspond to the isogyres and isochromates, respectively. The spectra are sufficiently separated from the origin of Fourier spectrum representing the DC content of the interference pattern. The spectra corresponding to the isochromates and isogyres are individually filtered and transformed to the laboratory coordinates by tracing back the steps through inverse transforms. Figure 7(b) shows the spectra used to obtain the intensity information regarding isochromates and Fig. 7(c) shows the spectra used to obtain the intensity information regarding isogyres shown in the top row of Fig. 4. To obtain the phase maps shown in the top row of Fig. 4, only the positive frequencies of the corresponding spectra shown in Fig. 7(b,c) are used. The schematic of the procedure followed to extract the isochromates and isogyres information from the conoscopic interference pattern is shown in Fig. 8.

## Additional Information

**How to cite this article**: Samlan, C. T. *et al*. Isogyres – Manifestation of Spin-orbit interaction in uniaxial crystal: A closed-fringe Fourier analysis of conoscopic interference. *Sci. Rep*. **6**, 33141; doi: 10.1038/srep33141 (2016).
